# Transapical Mitral Valve-in-Ring Replacement Using the Innovative System under 3-Dimensional Printing Guidance

**DOI:** 10.3390/jcdd10080339

**Published:** 2023-08-07

**Authors:** Yiwei Wang, Yu Mao, Mengen Zhai, Yanyan Ma, Lanlan Li, Yang Liu, Jian Yang

**Affiliations:** Department of Cardiovascular Surgery, Xijing Hospital, Air Force Medical University, Xi’an 710032, China; wangyiwei0911@hotmail.com (Y.W.); maoyu0704@126.com (Y.M.); zhaimengen@126.com (M.Z.); mayanyan930@126.com (Y.M.); lilanlan1124@126.com (L.L.)

**Keywords:** valve-in-ring, Mi-thos system, transcatheter mitral valve replacement, failed annuloplasty ring, 3-dimensional printing

## Abstract

Background: Transcatheter mitral valve-in-ring replacement (TMViR) is an emerging alternative for patients with recurrent mitral regurgitation (MR) after a prior failed annuloplasty ring. However, intraoperative common issues and complications remain to be addressed. Case summary: We describe the case of a 67-year-old male patient who underwent surgical mitral concomitant tricuspid annuloplasty repair 7 years ago who developed recurrent severe MR (New York Heart Association functional class IV). To avoid a high-risk surgical reoperation, we chose to perform a TMViR using an innovative dedicated device—the Mi-thos system—via a transapical approach. A patient-specific, 3-dimensional printed model was used to guide the procedure to avoid potential challenges. The procedure was performed successfully, and the patient exhibited symptomatic improvement. Conclusions: This case report highlights the first use of the innovative Mi-thos system in a TMViR procedure. The findings demonstrate the feasibility and safety of utilizing the Mi-thos system, guided by 3-dimensional printing technology, for patients who have experienced recurrent mitral regurgitation MR following a failed annuloplasty ring.

## 1. Introduction

Mitral regurgitation (MR) that occurs after a failed annuloplasty ring can have negative clinical consequences and effect long-term outcomes. In patients who are ineligible for a high-risk reoperation, interventional therapy for recurrent MR following surgical repair can be considered as an alternative option [1]. However, the current commercially available devices for percutaneous interventions are considered “off-label” and may have limitations when applied to the valve-in-ring concept [2]. Given the pathological structural complexity of the mitral valve (MV) as well as the risk of complications, there is a definite requirement for comprehensive preoperative assessment and simulation to determine equipment feasibility and ensure procedural success [2,3,4,5].

In recent years, the application of cardiovascular 3-dimensional (3D) printing technology has proven to be highly beneficial in facilitating precise preprocedural assessment and guidance for structural heart interventions [6,7,8,9,10]. We present the first case report of a transcatheter mitral valve-in-ring replacement (TMViR) procedure utilizing the innovative Mi-thos system (NewMed Medical Co., Ltd., Shanghai, China) via the transapical approach. The Mi-thos system is specifically designed as a dedicated device for transcatheter mitral valve replacement (TMVR). To enhance the planning process and evaluate potential intra- and postoperative risks, a preoperative patient-specific3D-printed model was constructed to simulate the key procedural steps.

## 2. Case Report

The patient is a 67-year-old male who underwent surgical mitral repair and tricuspid annuloplasty due to severe mitral regurgitation (MR) and moderate tricuspid regurgitation. The procedure involved the use of a 36 mm Edwards Physio annuloplasty ring (Edwards Lifesciences, Irvine, CA, USA). Three months ago, the patient was admitted to our department with fatigue, chest tightness, and exertional dyspnea (New York Heart Association functional class IV). Echocardiography showed recurrent severe MR (volume = 36.0 mL; V_max_ = 167 cm/s), and maximum transvalvular gradients were 13 mmHg. The left ventricle was dilated and showed a systolic ejection fraction of 46%.

**Figure 1:**
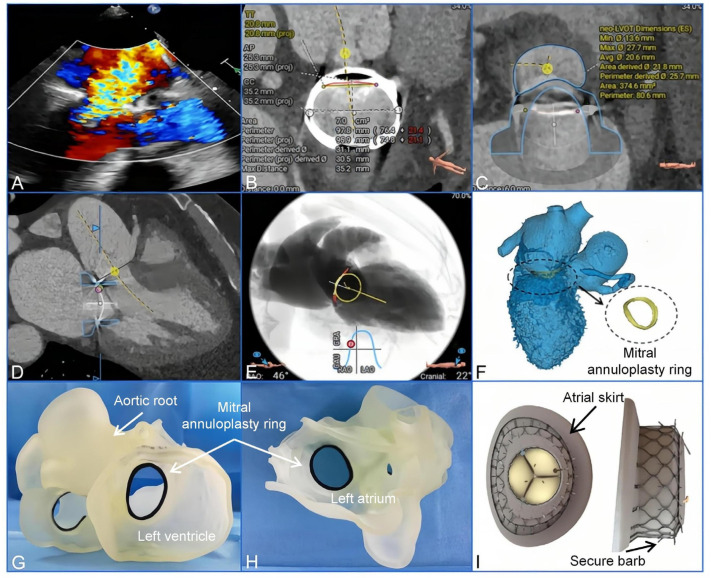
Preoperative imaging assessments and 3-dimensional-printed model reconstructions were used to formulate the procedural plan. (**A**) Preoperative echocardiography revealed severe mitral regurgitation. (**B**–**E**) Preoperative computed tomography angiography was used to access the mitral valve annulus and the left ventricular outflow tract. (**B**) The area of the mitral annuloplasty ring was 7.0 cm^2^. (**C**,**D**) The area of the neo-left ventricular outflow tract after implanting the bioprosthesis of the Mi-thos system was 374.6 cm^2^, and no obstruction occurred. (**E**) The projection angle of the released implanted valve was RAO46, CAU22. (**F**) Three-dimensional reconstruction and calculation of landing-zone structures were based on computed tomography angiography data. (**G**,**H**) The preoperative 3-dimensional printed model in the left atrial and the ventricular views. (**I**) With a D-shaped design, the bioprosthesis of the Mi-thos system fit the saddle-shaped mitral annulus.

A multidisciplinary cardiac team performed a meticulous evaluation of his transesophageal echocardiography (TEE) scans and computed tomography angiography scans and obtained the informed consent after having discussed all available treatment methods. Since the patient was highly symptomatic and at very high risk of secondary thoracotomy surgery, it was decided to perform the TMViR due to the possibility of the need for a high-risk surgical reoperation (based on Society of Thoracic Surgery score, 11.192%; European System for Cardiac Operative Risk Evaluation Score II, 8.3%) (Figure 1A–E). Meanwhile, a patient-specific3D-printed model was created using computed tomography angiography data. The image data, in Digital Imaging and Communications in Medicine format, were collected and processed using Materialise Mimics 21.0 software (Materialise, Leuven, Belgium). The software was used to segment the images and export them in the Standard Tessellation Language (STL) format. Post-processing of the STL files was performed using Materialise 3-Matic software (Leuven, Belgium, version 14.0). The final STL files were then imported into a Polyjet 850 multimaterial full-color 3D printer (Stratasys, Inc., Eden Prairie, MN, USA). The printer utilized materials with different flexibilities to accurately represent the structural characteristics of different parts, such as the annuloplasty ring and myocardial tissue (Figure 1F–H). Then, the procedures were simulated during the bench test. After its release, the unfolded bioprosthetic valve was observed clearly. The information obtained was used to evaluate and prognosticate major perioperative complications. Because the area of a 36 mm Physio ring was 7.0 cm^2^, and in addition, because the ring was D-shaped and semirigid and had a high risk of complications for the transcatheter aortic valve-in-ring concept, we decided to implant the Mi-thos system (Figure 1I) via a transapical approach.

**Figure 2:**
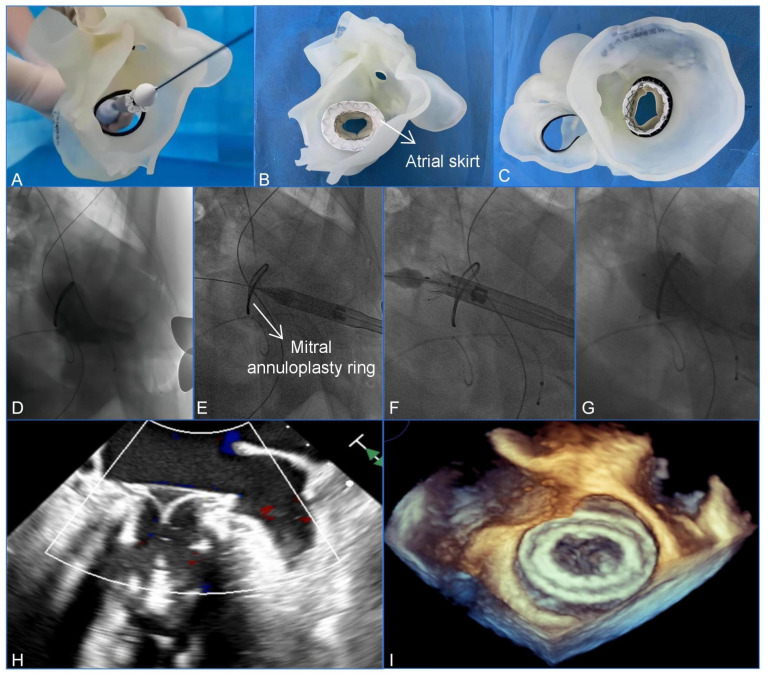
Preoperative assessment and simulation of and main procedural steps for transcatheter mitral valve-in-ring replacement. (**A**) Adjustment of coaxiality and of the release position. (**B**,**C**) The stent was fully unfolded and observed in the left atrial and the ventricular views. (**D**–**G**) The main steps of the procedure. (**D**) Fluoroscopy revealed severe mitral regurgitation. (**E**) The delivery system was advanced via the transapical approach. (**F**) Initial release of the stent. (**G**–**I**) After the stent was fully released, fluoroscopy, and transesophageal echocardiography revealed that the bioprosthesis was in a stable position and functioning well without paravalvular leak.

The procedure was conducted with the patient under general anesthesia and mechanical ventilation. Intraoperative guidance was provided using fluoroscopic images and TEE. A 4 cm left anterior mini-thoracotomy incision was made to access the puncture point at the apex of the left ventricle. Puncture and cannulation were carried out using 6F sheaths on both the right femoral artery and vein. Subsequently, a temporary pacemaker electrode was carefully inserted into the right ventricle through the vein sheath. At the same time, a 6F pigtail catheter was advanced from the right femoral artery to the left ventricle. We successfully obtained standard apical access and secured the entry site using a hexagonal suture. To optimize visibility of both the left ventricle and atrium, we made adjustments to achieve a cranial right-anterior-oblique angulation. Ventriculograms were performed to evaluate the MR and to assess the shape of the left ventricle and the mitral annuloplasty ring. Based on the preoperative assessment and simulation conducted using the patient-specific3D-printed model (Figure 2A–C), the assembly of a 33 mm Mi-thos bioprosthesis onto the delivery system was performed in vitro. To begin the procedure, an apical puncture was made, and a 6 Fr sheath was inserted. A 6F pigtail catheter, along with a J-tipped 0.035-inch guide wire, was then guided across the mitral surgical ring and into the left atrium. Following this, a 260 cm Lunderquist super stiff wire (Cook Medical Inc., Bloomington, IN, USA) was then used for exchange. The delivery system was inserted over the Lunderquist guide wire and advanced in a retrograde manner across the mitral annuloplasty ring via the transapical approach, ultimately reaching the left atrium. By retracting the outer sheath of the delivery system, the atrial skirt, which was specifically designed to fit the D-shaped mitral annulus, was released. It completely covered the mitral annulus and the annuloplasty ring, guided by TEE and fluoroscopy, while the ventricular portion of the device remained partially confined to the sheath. After confirming the position and alignment of the Mi-thos valve, the entire system was retracted and properly seated on the mitral annuloplasty ring. Subsequently, under rapid pacing at a rate of 140 per minute, the ventricular portion of the bioprosthesis was released and securely anchored to the native MV using secure barbs.

Immediately ventriculograms confirmed the excellent position and shape of the Mi-thos valve, with no evidence of transvalvular regurgitation, paravalvular leak (PVL), or left ventricular outflow tract obstruction (LVOTO) (the area of the neo-left ventricular outflow tract was 374.6 cm^2^). The valve’s position and function were further assessed using TEE, which indicated a mean pressure gradient of 3 mmHg (Figure 2D–I, Videos S1 and S2). Subsequently, the delivery system was carefully removed through the apex incision, and hemostasis was achieved using the apical strings that were previously placed. After closing the incision, the patient was successfully weaned off the ventilator and transferred to the cardiac intensive care unit. The postoperative recovery was smooth, and the patient was discharged in good clinical condition 5 days after the operation. At the time of discharge, the transthoracic echocardiogram examination confirmed the excellent position and function of the valve. Additionally, there was no evidence of any significant transvalvular or paravalvular regurgitation, as well as no LVOTO, and the maximum and mean gradients were measured at 6 mmHg and 2 mmHg, respectively. The patient’s symptoms had improved (NYHA functional class II).

## 3. Discussion

Despite advancements in surgical techniques and experience, the use of annuloplasty rings for various MV diseases still carries a high risk of recurrence. Additionally, redo MV operations have shown higher short-term mortality rates compared to initial operations, making them extremely risky for elderly and high-risk patients with recurrent MR [3]. Meanwhile, TMVR procedures have been performed to offer a potential solution for patients with failed annuloplasty rings at high or extreme risk for surgical reoperation and showed excellent clinical results. However, there are several challenges associated with this approach. The TMViR technique typically involves the use of the commercially available SAPIEN platform from Edwards Lifesciences [4]. However, the use of rigid and eccentric surgical rings as landing zones for cylindrical transcatheter heart valves can be problematic and may hinder optimal device expansion. In cases where larger ring areas are involved, the options for prostheses in TMViR procedures are limited. This limitation can lead to high-risk complications such as valve migration, embolization, PVL, and even LVOTO [2]. Therefore, it is crucial to have accurate preoperative assessment and simulation, as well as dedicated devices, to ensure the success of TMViR.

In recent years, there have been promising results from clinical trials of various TMVR devices [11,12]. However, these devices are not yet widely available for the large population of patients with MV disease, and their integration into routine clinical practice remains limited. These can be attributed to the unique structure of the MV, which also presents challenges in preoperative evaluation and selection of appropriate TMVR devices. Given the intricacies of the MV, which is a spatial 3-dimensional structure with diverse lesion patterns and dynamic movement during the cardiac cycle, the conventional evaluation methods have their limitations in accurately assessing the severity of MV conditions [2,3,4,5]. Additionally, the mitral position may exhibit heterogenous implants after surgical intervention, which can pose challenges for the successful implantation of new devices. Moreover, this heterogeneity adds to the complexity of evaluating the feasibility of the procedure and assessing potential postoperative complications. Therefore, careful consideration and planning are crucial to ensure the optimal outcome of the procedure in such cases.

The benefits of using 3D printing in structural cardiac interventions have been well-documented. To address these challenges, preoperative evaluation and in vitro simulation guided by 3D printing technology can be an effective solution [6]. This method offers a valuable solution to address the limitations of evaluating complex MV, enabling a more comprehensive and precise assessment of anatomical structures. Additionally, 3D printing can guide device selection and evaluate device–bioprosthesis interactions and potential complications [7,8,9]. Mao et al. [8] utilized 3D printing to restore the patient’s degenerative mitral bioprosthetic structure, facilitating evaluation and simulation prior to the valve-in-valve procedure. This approach allowed for accurate selection of the appropriate size of the balloon-expandable valve and ensured optimal device positioning, resulting in satisfactory clinical outcomes. Ginty et al. [9] also developed a dynamic MV model to simulate transcatheter mitral valve repair. The findings demonstrated that the dynamic model successfully replicated the hemodynamic characteristics of both normal and pathological conditions of the MV, offering potential support for enhanced clinical diagnosis and treatment.

We present a case of a patient who underwent TMViR. A patient-specific, 3D-printed model was utilized to gain valuable insights into the shape of the landing zone, facilitating discussions regarding valve sizing and determining the optimal position for the valve-in-ring procedure [6,7,8,9,10]. Moreover, the model enabled simulation of the main procedural steps and prediction of potential intraoperative complications, and the utilization of 3D printing technology allows us to thoroughly explore the unique properties of new devices and significantly reduces the learning curve associated with their implementation. This approach proves to be instrumental in enhancing our understanding of the device and its compatibility with individual patients, thereby improving the overall success rate of TMVR procedures [7,8,10]. Our findings revealed that cylindrical transcatheter heart valves implanted into the semi-rigid and large-sized mitral surgical ring resulted in significant PVL, as well as the potential risk of valve displacement or embolization. Based on careful evaluation and consideration, we made the informed decision to select the Mi-thos system for the valve-in-ring procedure. This advanced and dedicated TMVR device offers innovative features and capabilities that align with the specific needs and requirements of the patient with MV diseases.

The system under consideration is composed a self-expanding device that consists of cross-linked bovine pericardial tissue leaflets securely mounted within a nitinol frame and a 36-F transapical delivery system. This frame incorporates a modified and enhanced version of the original conceptual structure, optimizing its performance and functionality [13]. Significantly, this stent features a dual-frame design that combines a D-shaped outer stent with a circular inner stent. This innovative configuration is specifically tailored to accommodate the saddle-shaped mitral valve anatomy and promote optimal leaflet opening. The bioprosthesis is equipped with a D-shaped atrial skirt that is securely connected to the atrial end of the fixation ring. This design feature enhances support and stability, promoting proper positioning and reducing the risk of regurgitation around the valve. Additionally, the device incorporates a unique anchoring mechanism consisting of three layers of small barbs strategically arranged around the ventricular portion of the outer frame. These hangnails effectively capture the mitral annulus and leaflet, further enhancing fixation and stability. Moreover, the valve design includes an S-shaped spring connecting the valve body and skirt, which ensures adequate radial force of the valve body and provides sufficient compliance on the atrial side of the skirt. These carefully engineered features work together to ensure secure fixation and stability within the mitral valve structure. Furthermore, the Mi-thos system offers the advantage of being retrievable and repositionable [13]. These structural elements and features effectively eliminate PVL and prevent retrograde migration.

Before performing the procedure, we utilized a patient-specific, 3D-printed model to simulate the implantation of the Mi-thos system for TMViR. The simulation revealed that the larger D-shaped atrial skirt of the device can provide coverage over the mitral annuloplasty ring, effectively preventing PVL. Additionally, the presence of barbs on the stent allows for enhanced anchoring with the surgical annulus. Moreover, the use of a shorter stent reduces the risk of LVOTO. Ultimately, we successfully performed TMViR via the transapical approach using a 33 mm Mi-thos system into a 36 mm mitral surgical ring, guided by 3D printing technology, and achieved favorable clinical outcomes.

## 4. Conclusions

This case report highlights the successful utilization of the Mi-thos system in a TMVR procedure, specifically following the failed mitral annuloplasty ring via the transapical approach. The remarkable ease of control and intraoperative patient stabilization demonstrated in this case signifies the potential need of minimally invasive methods in carefully selected cases. Furthermore, one of the key advantages of this device is its ability to minimize complications associated with TMVR procedures. The incorporation of 3D printing guidance in surgical planning enhances the feasibility and safety of TMViR, providing a valuable tool for developing effective surgical strategies.

## Data Availability

Data were uploaded as suggested by Data Availability Statements in section “MDPI Research Data Policies”.

## Supplementary Materials

The following supporting information can be downloaded at: https://www.mdpi.com/article/10.3390/jcdd10080339/s1. Video S1: Fluoroscopy image showed the bio-prosthesis functional well without mitral regurgitation; Video S2: TEE showed the bio-prosthesis functional well without mitral regurgitation.
